# Modeling the evolution of sensitive periods

**DOI:** 10.1016/j.dcn.2019.100715

**Published:** 2019-11-12

**Authors:** Willem E. Frankenhuis, Nicole Walasek

**Affiliations:** Behavioural Science Institute, Radboud University, the Netherlands

**Keywords:** Sensitive periods, Critical periods, Evolution, Plasticity, Development, Formal modeling

## Abstract

In the past decade, there has been monumental progress in our understanding of the neurobiological basis of sensitive periods. Little is known, however, about the evolution of sensitive periods. Recent studies have started to address this gap. Biologists have built mathematical models exploring the environmental conditions in which sensitive periods are likely to evolve. These models investigate how mechanisms of plasticity can respond optimally to experience during an individual’s lifetime. This paper discusses the central tenets, insights, and predictions of these models, in relation to empirical work on humans and other animals. We also discuss which future models are needed to improve the bridge between theory and data, advancing their synergy. Our paper is written in an accessible manner and for a broad audience. We hope our work will contribute to recently emerging connections between the fields of developmental neuroscience and evolutionary biology.

## 1 Introduction

### 1.1 Finding common ground

Sensitive periods are widely studied across the social and biological sciences. The term is used in different ways in different disciplines. In this paper, we define a ‘sensitive period’ as a time period (or life stage) in which experience shapes a trait to a larger extent than the same experience does in other time periods (Fawcett and Frankenhuis, 2015). This definition is intentionally broad: the effects of experience are not necessarily limited to a sensitive period, and phenotypes developed during a sensitive period might be modifiable by later experience. Further, the definition is agnostic about mechanism; it concerns only the impact of experience on phenotype, not the mechanisms that implement this relation. Such generality has pros and cons. Pros are: this definition can be applied across species (from plants, to animals without brains, to homo sapiens) and to diverse phenomena (e.g., early programming, plasticity declining with age, adolescence offering new opportunities for adaptation). Cons are: when a general definition is applied to any particular species, it ignores mechanisms known to implement sensitive periods in this particular species (e.g., experience-*expectant* and experience-*dependent* plasticity in humans; Gabard-Durnam and McLaughlin, 2019; Galván, 2010; Greenough et al., 1987; Johnson, 2005).

Developmental neuroscientists often study sensitive periods using the framework of experience-*expectant* and experience-*dependent* plasticity. The former is plasticity that integrates “environmental information that is ubiquitous in the environment and common to all species members” (Greenough et al., 1987, p. 539); it involves neural mechanisms that come prepared for incorporating specific information (e.g., invariants in perceptual input). The latter, in contrast, is plasticity that integrates “environmental information that is idiosyncratic, or unique to the individual” (idem); it involves active formation of new synaptic connections in response to specific situations, which differ between individuals. This distinction captures an impressive array of processes in a variety of species, and has enabled tremendous progress in our understanding of the neurobiological mechanisms of plasticity. Moreover, subsequent elaborations of the distinction – which incorporate more refined descriptions of, for instance, the effects of timing and dose of experience (Dunn et al., 2019; Gabard-Durnam and McLaughlin, 2019) – are even better able to accommodate heterogeneity in contemporary data. We fully acknowledge the merit of this framework. Yet, we also agree with the scholars who originally developed this framework that it paints “a much more straightforward picture (…) than probably exists” (p. 551). In particular, the framework has limitations when applied across the full tree of life.

The framework does not capture all classes of plasticity particularly well. For instance, polyphenic traits are traits where multiple, discrete phenotypes emerge from a single genotype, depending on environmental conditions. In many reptiles, variations in nest temperature determine sex (whether an organism becomes male or female). In many insects, temperature, photoperiod, or nutrition determines the caste of an individual (e.g., a larvae can develop into a queen, worker, or soldier). In many crustaceans, exposure to chemicals released by predators induces the development of defensive armor (Gilbert, 2003). Such polyphenisms respond to environmental information that is not common to all species members, nor unique to an individual. Rather, all members of a species have evolved to ‘expect’ different states of the environment, and are ‘prepared’ to develop a range of different phenotypes, depending on environmental or somatic conditions. The same logic applies to developmental mechanisms that are evolutionarily prepared to produce continuous phenotypic variation (e.g., adaptive calibration of the rate of maturation in response to nutritional condition).

Polyphenic traits have some characteristics of experience-*expectant* plasticity, some of experience-*dependent* plasticity, and some that fit neither class well. All species members ‘expect’ particular experiences and are ‘prepared’ to respond to them. However, these experiences differ between individuals, frequently in non-idiosyncratic ways (e.g., in each generation, a predictable proportion of the population is exposed to each type of experience). Moreover, in some cases, the impact of experience is restricted to a single time window; in others, there are several time windows. In some cases, these windows are well delineated; in others, their onset and offset are more gradual. In some cases, time windows are neatly ordered; in others, their ordering is more variable. In some species, the effects of experience are irreversible; in others, they can be reversed (e.g., some fish can switch sex multiple times, including sex-specific behavioral repertoires, depending on social hierarchy), albeit perhaps more easily in some life stages than others. In terms of mechanism, some of these responses depend on neural overproduction and pruning, but many do not (e.g., the development of defensive armor in crustaceans).

Nature rarely comes only in two kinds. More often, it presents a smorgasbord. In such cases, dichotomous frameworks can be extremely powerful, but not necessarily for all purposes. In this paper, we need a broad definition that describes changes in plasticity across ontogeny in a wide range of species. In addition, we want our definition to exclude cases where plasticity does not change across ontogeny. We have already noted that our definition also has limitations; it is agnostic about mechanism. When studying species that fit experience-*expectant* and experience-*dependent* plasticity, researchers may prefer to use those terms.

### 1.2 Why do sensitive periods exist?

Why are organisms not *Darwinian demons*, capable of adjusting their phenotypes with equal ease to new conditions throughout their lifetimes (Law, 1979)? In reality there is variation in plasticity (i.e., the ability to tailor development based on experience) between different species, between individuals, and even between different brain systems within a single individual. For instance, some bird species are only able to learn new songs in their first weeks, while others retain this ability throughout their lives (Beecher, and Brenowitz, 2005). After being adopted, some children adjust better than others to the new conditions (Ellis et al., 2011). And, different brain systems within a person may adjust to new conditions at different rates (Zeanah et al., 2011).

In the past decade, there has been formidable progress in our understanding of the mechanisms that determine changes in plasticity over the life course (Takesian and Hensch, 2013). It is now possible to modify aspects of sensitive periods (such as their timing and duration) for a range of neurobiological systems in different species, through experiential or pharmacological manipulation. This research truly has applied potential. For instance, it can inform interventions that erase neural signatures of trauma (Hensch and Bilimoria, 2012). It also raises important ethical questions; for instance, whether it is ever ethical to apply such techniques to humans, and if so at what age and on which grounds. Despite great progress in our understanding of *how* sensitive periods work, we know little about *why* sensitive periods exist. Specifically, we know little about the conditions that favor the evolution of sensitive periods, about which sensitive periods are adaptive and which ones are not; and if adaptive, what a sensitive period’s function is. There are plausible hypotheses about specific observations, but there is no unifying theory.

Fortunately, a unifying framework is starting to emerge in biology. In the past decade, a set of formal (i.e., mathematical) models has emerged exploring the evolution of sensitive periods. These models examine how mechanisms of plasticity can respond in fitness-enhancing ways to experience during an individual’s lifetime (see Section 2.2); and, as a consequence, produce variation in plasticity between species, individuals, and systems within the brain. Biologists acknowledge that not all plastic responses, nor all variations in plasticity, are adaptive. Yet, they explore ‘optimal’ developmental responses in different conditions. The reason is epistemological: in order to know which variation is adaptive, we need theory predicting what animals ought to do ‘if’ they are responding adaptively (Frankenhuis et al., 2013, 2018). If predictions match observations, we find some support for our model and gain new insight. If predictions and observations do not match, we should modify our model, for instance, by incorporating constraints on animals’ abilities to sample and use of information available in their environment in an optimal way (Gigerenzer et al., 1999; Marcus, 2009). This optimality approach is already used widely in different subfields of biology (e.g., functional morphology, behavioral ecology) and in cognitive neuroscience as well (see Section 2.1).

We do not discuss formal models in detail here (see Fawcett and Frankenhuis, 2015; Frankenhuis and Fraley, 2017). Rather, we will describe their central tenets, insights they provide, their predictions, and a selective review of empirical research on humans and other animals. In addition, we discuss which future models are needed to improve the bridge between theory and data, advancing their synergy.

## 2 Evolutionary models of sensitive periods

### 2.1 The value of modeling

Formal models have several advantages over natural language. Natural language is often more ambiguous than mathematics, and inferences – for instance, from premises to predictions – based on human reasoning are more fallible (e.g., subject to confirmation bias) than a mathematical analysis. Almost 200 years ago, Darwin recognized the value of mathematics. He wrote in his autobiography: “I have deeply regretted that I did not proceed far enough at least to understand something of the great leading principles of mathematics; for men thus endowed seem to have an extra sense” (1828–1831). As with natural language theories, the utility of any particular model will depend on the validity of its assumptions (do these match the phenomenon of interest), the rigor of its analysis (exploring all of the relevant conditions), and the interpretation of results (drawing reasonable conclusions about the world).

Models, like maps, focus our attention on some factors and processes, while leaving out others (Epstein, 2008; Smaldino, 2017). In cognitive neuroscience, models focus on neurocognitive processes and their outcomes (van den Bos and Eppinger, 2016). In evolutionary biology, models focus on evolutionary processes (e.g., natural selection, mutation) and their outcomes. Models in cognitive neuroscience are often fitted to data (such as decisions, reaction times, and brain activity) with the goal to better understand the processes generating these data. By contrast, evolutionary models often start with general axioms (e.g., natural selection favors adaptive mechanisms), make additional assumptions about a phenomenon (e.g., plastic mechanisms tailor phenotypes to local conditions), and explore which mechanisms are favored, depending on environmental conditions (Frankenhuis et al., 2013, 2018).

Cognitive neuroscientists use formal models to explore questions about causal mechanisms at a *proximate* level. Evolutionary biologists use models to explore questions about evolutionary selection pressures at an *ultimate* level. For instance: If environmental conditions fluctuate at a particular rate, should natural selection favor plasticity or not? If plasticity is favored, should its level be uniform or variable across ontogeny? If variable, should we expect the onset and offset of enhanced plasticity to be punctuated or gradual? Is there one peak or multiple ones? Although evolutionary models do not directly provide insight into neurobiological mechanisms, they do offer hypotheses about the factors and processes that influence levels of plasticity (see Section 3). However, prediction is not the only goal of models. Models also help to organize existing observations, for instance, by explaining the adaptive function (or lack thereof) of known mechanisms. Proximate and ultimate explanations are not in opposition to each other, but mutually compatible; they exist at different levels. A biologist who has achieved a complete understanding of the neurobiological mechanisms of plasticity in, say, a soapberry bug, might still wonder: ‘What evolutionary selection pressures have favored plasticity in this species?’

A study of soapberry bugs – a half-inch-long, seed-eating insect – illustrates how plasticity can evolve. In Oklahoma, harsh weather conditions (e.g., storms) randomly kill subsets of individuals, and so soapberry bugs are exposed to a variable sex ratio (the ratio of females to males). There, males have evolved the ability to adjust their levels of mate guarding. When there are many rivals, they guard their current mate. When there are few rivals, they search for new mates. In Florida, by contrast, the weather is not harsh and so the sex ratio is stable over time. There, males have not evolved plasticity in their level of mate guarding; that is, males in Florida always guard the same amount. If these males are artificially exposed to varying sex ratios in the lab, they do not adjust their levels of mate guarding (Carroll and Corneli, 1995). This study shows that plasticity is a target of natural selection. Although this study did not examine whether there are sensitive periods in the development of mate guarding, formal models could be developed that explore at what life stages soapberry bugs should sample the local sex ratio, for how long they should sample, and how observations of the local sex ratio should affect their behavior.

Evolutionary models thus help to explain, at an ultimate level, ‘why’ different mechanisms have evolved in different species and traits; but not, at a proximate level, ‘how’ these mechanisms work. These models do, however, offer predictions about ‘how’ animals should respond to experience at a behavioral level. Such predictions offer insight into proximate-level, developmental processes, even when these predictions do not tell us ‘how’ behavior is accomplished (e.g., prolong plasticity if experience is too noisy to infer the statistical structure of the environment). Until biologists and neuroscientists achieve a complete understanding of mechanisms, therefore, evolutionary models can do more than just explain variation between species and traits; they can also help to uncover the factors and processes that influence the onset, duration, and offset of plasticity across ontogeny.

### 2.2 Evolutionary modeling of sensitive periods

All phenotypes, even those shared among all members of a species, result from developmental processes. It follows that natural selection can only influence phenotypes by shaping developmental systems; that is, the array of causal factors and processes that construct phenotypes (Barrett, 2015; Frankenhuis et al., 2013). The modeler wants to understand what evolutionary pressures, across generations, result in mechanisms that produce sensitive periods, within generations, based on experience.

Evolutionary models of sensitive periods do not start out assuming a sensitive period. Rather, such a period might emerge as the outcome favored by natural selection; that is, the outcome that maximizes biological ‘fitness’. Psychologists often use the term fitness to denote individual survival and reproduction. Biologists, however, typically use the term to refer to the reproductive success of developmental systems (or mechanisms, strategies, genotypes). These systems generate distributions of phenotypes (individuals), which might pass on the developmental system by reproducing. Thus, offspring inherit developmental systems from their parents. The fitness of a developmental system, then, depends on the extent to which the individuals it generates produce more offspring than those produced by other systems. From this viewpoint, the adaptive value of a sensitive period depends not on whether any particular individual benefits from it. What matters, rather, is whether the system achieves high fitness relative to other systems, *because* it generates phenotypes that are more affected by experience at certain times of life than others (e.g., early in ontogeny). Section 4.2 discusses different ways to quantify the impact of experience on phenotype.

All developmental systems include both genotypic and environmental factors and processes. However, the roles of these components in the production of phenotypes differ between developmental systems (Barrett, 2015; Bjorklund and Ellis, 2014; Frankenhuis et al., 2013; Gottlieb, 1991; Lickliter and Honeycutt, 2003; Oyama et al., 2001; Tooby et al., 2003). Some systems use aspects of their environments that are shared among all species members (e.g., invariants in the visual environment used to construct perceptual abilities). Others use aspects of their environments that vary between species members (e.g., polyphenisms). Both types of systems are common in nature, and both may exhibit sensitive periods. However, all evolutionary models of sensitive periods (that we are aware of) have explored systems that are exposed to environmental variation between generations, within generations between individuals, or both. There is, therefore, clearly a need for models of the evolution of sensitive periods in neural systems that are adapted to environmental invariants. Here, we restrict our discussion to existing models. The question addressed by these models is whether experience should differently affect development, depending on its timing, dose, the information it provides, and so on.

To be able to explore these kinds of effects, a model needs to include two or more time periods in which organisms are able to access ‘cues’ that can shape their phenotypes (Frankenhuis et al., 2018). A cue is an observation that provides information (i.e., reduces uncertainty), either about the environment (e.g., safe or dangerous) or about the organism itself (e.g., its somatic condition). Cues are often imperfect (e.g., there may be smoke but no fire). The reliability of a cue depends on the extent to which it discriminates between different states of the environment or states of the organism. A cue has high reliability if it is much more likely to occur in certain states of the world than others (e.g., violence is more likely to occur in poor than in rich neighborhoods). A cue has low reliability if it is almost equally likely to occur in different states of the world (e.g., seeing a person lock their house may be about equally likely in poor and rich neighborhoods). Many species use cues to infer their current conditions (e.g., the level of danger), and some species also use cues to predict their (likely) future conditions. As we discuss below, the reliability of cues might affect the optimal level of plasticity, because this optimal level might depend on the extent to which an organism ‘knows’ (has information about) what the current conditions are and how likely these conditions are to change or remain the same.

Evolutionary models usually conceptualize development as a sequential decision-making process. These models often describe the ‘state’ of an organism, which determines the decisions it makes, in terms of two components: estimates of the environment (e.g., safe or dangerous) and phenotypic condition (e.g., nutritional reserves). The model then computes, for every possible state of an organism, which decision maximizes the fitness of its mechanisms. We distinguish between ‘mechanism’ (or strategies, genotypes) and ‘organism’ here too because, as noted, decisions that are optimal for a mechanism might produce outcomes that are actually detrimental for a subset of individuals (Frankenhuis and Del Giudice, 2012). For instance, in winner-takes-all mating systems (e.g., the alpha has many more babies than other group members, as in elephant seals), it may be adaptive for developmental mechanisms to produce aggressive animals that vie for the top rank in the social hierarchy. Such mechanisms may have higher fitness than alternative mechanisms that produce less aggressive individuals. Fighting is stressful and some animals will die. Yet, fighters may be maximizing the fitness of the mechanisms that created them.

In cognitive modeling, a prior is the estimate of an individual at the beginning of a decision problem (e.g., whether or not to wait for a reward). This estimate usually has its source within the individual’s lifetime (Dunlap and Stephens, 2016). It is based on personal experience (e.g., past promises were broken) or learned socially (e.g., people say future rewards are unreliable). In evolutionary models, in contrast, the prior does not necessarily represent psychological knowledge. Rather, it is an adaptation of a developmental system to the distribution of environments experienced by a lineage over evolutionary time. For instance, if a species was consistently exposed to high levels of predation, it may embody this statistical regularity by building anti-predator defenses by default, unless it receives strong evidence (contradicting the prior) that the current environment is actually safe. In evolutionary models, organisms may inherit their priors from their distant ancestors (e.g., via genes), from their immediate ancestors (e.g., via parental effects, epigenetic factors), or a combination of both (Dall et al., 2005, 2015; Mangel, 1990; McNamara et al., 2006; Pfab et al., 2016; Stamps and Frankenhuis, 2016; Stamps and Krishnan, 2014a, 2014b; Trimmer et al., 2011; Uller et al., 2015). Organisms update their priors based on the cues they sample during their lifetimes – often in a Bayesian fashion, the optimal way of updating – while making decisions that affect their phenotypes. These decisions and their phenotypic consequences illuminate the evolution of sensitive periods.

## 3 Insights offered by evolutionary models of sensitive periods

Evolutionary models of sensitive periods have produced a variety of insights. We do not provide an exhaustive discussion of these insights here. Rather, we focus on two insights that are particularly relevant to developmental cognitive neuroscience. Readers who wish to read more may consider the following resources (Fawcett and Frankenhuis, 2015; Frankenhuis and Fraley, 2017; Stamps and Krishnan, 2017).

### 3.1 Plasticity depends on information about the current environment

The first insight is that plasticity often depends on the extent to which the prior and cue reliability, that an animal is adapted to, provide information (reduce uncertainty) about the current state of the environment. Animals that are adapted to more uncertainty about current or future conditions – for instance, because their lineage evolved in diverse environments, or because cues have low reliability) might benefit from having greater plasticity. In many cases, the amount of information an animal is adapted to increases over its lifetime, because the animal learns about its environment; as a consequence, its plasticity might decline. This finding emerges in many models, and within models across a broad range of parameter combinations.

This finding fits with empirical research showing that sensitive periods in *experience-expectant plasticity* might be prolonged if: (i) animals are deprived of cues (Hensch, 2004; Knudsen, 2005; Michel and Tyler, 2005); (ii) animals process noisy cues (Chang and Merzenich, 2003); (iii) or cues that are gradually changing (Bateson and Martin, 1999; Bolhuis, 1991); or (iv) perceptual systems offer the brain unstable inputs, possibly because they are developing or are disrupted (Thomas and Johnson, 2008). For instance, in zebra finches, the absence of tutors extends the sensitive period for song learning (Kelly et al., 2018), with greater numbers of new neurons being added to high vocal center (Wilbrecht et al., 2006). The absence of face input prevents perceptual narrowing in Japanese macaques (Sugita, 2008). Exposing rat pups to a stream of white noise delays their auditory specialization (Chang and Merzenich, 2003). However, deprivation does not *merely* prolong sensitive periods. In humans, for instance, it can accelerate synaptic pruning and limit myelination, reducing cortical thickness and white matter integrity (McLaughlin et al., 2017); and related, in non-human primates, result in neural disuse and inefficient processing (Scott et al., 2007). In general, the effects of adverse experience on the brain are complex and diverse, because they result from a multitude of processes (Galván, 2010; Gabard-Durnam and McLaughlin, 2019). We argue that one such process is the rate at which the brain is able to infer the statistical structure of the environment.

The common denominator is that the animal lacks access to reliable cues about its environment. As Bateson and Martin (1999) noted: “processes that bring the sensitive period to an end are related to the gathering of crucial information and, except in extreme cases, do not shut down until that information has been gathered” (p. 162). However, as we discussed in Section 2, the fit an animal achieves with its environment depends not only on the cues it collects, but also on the distribution of environments its lineage has adapted to (its evolved prior). The extent to which a given cue shifts this prior depends on its variance as well as the extent of agreement between prior and cue (Stamps and Frankenhuis, 2016). If a prior has more variance (more uncertainty about the state of the environment), a given cue shifts an estimate more than when it has less variance. And, the more a cue disagrees with the prior, the more the prior shifts.

Evolutionary modeling accordingly predicts, all else being equal, that if animals who have different priors are exposed to the same cue, those whose priors and cue are in agreement change their phenotypes less than those whose prior and cue disagree (Stamps and Frankenhuis, 2016). Biologists have recently tested this novel prediction in fruit flies (Stamps et al., 2018). They first showed that larvae vary in the extent to which they are attracted to the odor of ethyl acetate (a fruity smelling liquid); some flies had positive priors about ethyl acetate, others had negative priors. Then they showed that flies that had positive priors changed their behaviors more after an aversive training regime (experimental exposure to a negative cue) than flies with negative priors. So, the extent of phenotypic change depended on the convergence between prior and cue.

By hypothesis, individual variation in the duration of sensitive periods in humans may also depend on the agreement between priors and cues. If two individuals who have different priors are exposed to the same cues, the individual whose prior and cues agree more might lose their plasticity earlier. As priors are inherited from parents (e.g., via the genome or epigenome), we might expect children whose environment matches that of their parents to have shorter sensitive periods than children who develop in a different environment than their parents. Similarly, we might expect individuals who have more consistent experiences (e.g., all safe cues versus some safe cues and some danger cues), or more reliable cues (e.g., extreme experiences that occur only in extreme conditions) to reduce their uncertainty faster, and hence lose their plasticity earlier, than individuals who have less consistent experiences or who sample less reliable cues (Frankenhuis and Panchanathan, 2011a, 2011b; Panchanathan and Frankenhuis, 2016). To our knowledge, these hypotheses have not been tested in humans. The results of such tests would be of great interest to researchers in the field of ‘differential susceptibility,’ who study the developmental emerge of individual differences in plasticity (Ellis et al., 2011).

### 3.2 Predicting future environmental conditions or one’s own future health?

The second insight concerns both *why* sensitive periods exist and *which* factors may shape development during a sensitive period. A widespread view in clinical and developmental science is that it is adaptive for organisms to tailor their developmental trajectories based on early-life cues, because these cues predict future environmental conditions (Belsky et al., 1991; Ellis et al., 2009). For instance, if early conditions are harsh, this may provide a ‘weather forecast’ about likely future conditions, to which an organism can adapt (e.g., by reproducing at a younger age). Such ‘external predictive adaptive responses’ (or external PAR) have been demonstrated in some short-lived animals. For instance, in gravid field crickets, if a mother is exposed to a predatory spider, her offspring will develop anti-predator responses that increase their survival in an environment containing such a spider (Storm and Lima, 2010). In this case, the cue reliability is high (the mother has directly perceived the spider), and the stability of environmental conditions is likely to be high, as the offspring face the predation threat only a few days after receiving the prenatal cue, and in the same location. Similarly, there are compelling demonstrations of external PARs in plants where seed dispersal is limited, and therefore, offspring are likely to develop in the same light patch as their parents did (Galloway and Etterson, 2007). In the external PAR model, a sensitive period early in human development is thought to be adaptive because individuals benefit from preparing for future environmental conditions, based on their assay of conditions early in life; and in particular, estimates of environmental harshness and unpredictability (Ellis et al., 2009).

Recent evolutionary modeling shows that the evolution of external PAR in long-lived animals, such as humans, requires that environmental conditions are very highly autocorrelated and that cues very accurately reflect current environmental conditions (Nettle et al., 2013; Rickard et al., 2014; for discussion, see Del Giudice, 2014a). Only then do organisms benefit from relying on their early-life experiences. In other cases, they are better off tailoring their phenotypes to their evolved priors, and ignoring cues. This result has led to the internal PAR model. In this model, individuals benefit from tailoring their phenotypes based on adverse early experiences because these experience lead to somatic damage that shortens individuals’ expected lifespan. Thus, rather than adapting to future environmental conditions (a weather forecast), individuals are adapting to their own future somatic decline; that is, the weathering of their own bodies (a weathering forecast) (Geronimus et al., 2006).

Unlike the external PAR model, the internal PAR model does not require highly reliable cues to current conditions and high environmental autocorrelation. It does, however, require earlier somatic states to be correlated with later ones, such that the impact of early-life experience on adult internal state is substantial (Nettle et al., 2013). Somatic autocorrelation is known to exist in many species. For instance, telomeres, i.e., the protective “caps” on the end of chromosomes, appear to be markers of life’s insults (Blackburn et al., 2015), in that they are affected by exposures to stress and adversity; although a recent meta-analysis suggests this relation is less strong than previously thought (Pepper et al., 2018). Moreover, new empirical work suggests it may exist only for specific types of adversity exposures (e.g., threat) and not for others (e.g., deprivation) (Sumner et al., 2019). Telomeres shortened early in life tend to remain short for the rest of life, and short telomeres predict worse future health and longevity in humans (Bakaysa et al., 2007; Kimura et al., 2008; Njajou et al., 2009). Whether it is mediated by telomeres, oxidative stress, or a different factor, an internal PAR requires somatic autocorrelation, but not environmental autocorrelation.

Although the internal and external PAR models both expect a correlation between early adversity and reproductive development, these models make different predictions about the role of somatic health (Rickard et al., 2014). Whereas the external PAR model predicts worse health *as a consequence* of prioritizing investment in early reproduction (e.g., at the expense of somatic maintenance), the internal PAR model predicts that health *mediates* the relation between early adversity and reproductive development. Several studies have tested these predictions. Some of these studies found support for both models in a single dataset. For instance, a longitudinal study in the United States revealed that after controlling for health, early-life adversity predicts greater adolescent risk taking, problematic functioning, and earlier age of menarche in girls; with health mediating the relation between the early environment and adolescent behavior (Hartman et al., 2017; for related results, see Chua et al., 2017).

Other studies have found support for the internal PAR model by showing correlations between early somatic condition and reproductive outcomes, even if early somatic state is not correlated with early environmental stress, or after controlling for such stress. For example, British women who had a life-expectancy-reducing childhood disease were more likely to have their first child at a younger age than women without such a disease, even if their illness was not correlated with indicators of environmental stress, such as low socioeconomic status or father absence (Waynforth, 2012; for related results, see Brumbach et al., 2009; Hill et al., 2016; Valencia and Cromer, 2000). This finding highlights the importance of the internal-external PAR distinction: only the internal PAR model predicts that chronic illness early in life should have a lasting impact on the pace of life history development (i.e., there exists a sensitive period effect); which, the evidence to date suggests, it does.

The internal PAR model has also received support in various non-human animal species, such as reindeer and rhesus macaques (Berghänel et al., 2016; Douhard et al., 2016). These studies suggest the existence of internal PAR in wild long-lived animals that inhabit ecologies that are too unpredictable for external PARs to evolve. These studies have actually measured environmental statistics that have impact on the fitness of animals (e.g., year-to-year predictability of food availability) and have shown that environmental conditions in infancy and childhood are hardly, if at all, predictive of conditions in adolescence and adulthood. Yet, these animals accelerate their reproductive development if they experienced early-life adversity. For instance, macaques that inhabit Southeast Asian forests experience highly unpredictable food availability, so that it is not possible to predict food availability in adolescence and adulthood based on food availability early in life. Nonetheless, in this species, infants whose energy intake is reduced early in life as a result of their mother’s physiological stress have accelerated growth, consistent with their gearing up for earlier reproduction (Berghänel et al., 2016). Such findings are difficult to reconcile with the external PAR model, but are consistent with the internal PAR model. In short, the internal PAR model is not only interesting because it proposed a new explanation for the association between early adversity and reproductive development; it has also generated new empirical knowledge by inspiring research showing that, in some animals, health mediates developmental programming effects. It is an example of an ultimate-level hypothesis contributing to novel insights about proximate mechanisms.

## 4 Sensitive periods in adolescence and other life stages

Evolutionary models have shed light on the conditions favoring sensitive periods early in life. However, there may be sensitive periods in other life stages as well, such as middle childhood (Del Giudice, 2014b; Del Giudice and Belsky, 2011) and adolescence (Blakemore and Mills, 2014; Dahl, 2004; DePasquale et al., 2018; Fuhrmann et al., 2015; Sachser et al., 2018). Some of these sensitive periods might be adaptive. Few formal models, however, have explored the evolution of ‘mid-ontogeny’ sensitive periods. In this section, we discuss initial steps towards such models.

### 4.1 Adaptive reasons for sensitive periods

In a theoretical paper, Fawcett and Frankenhuis (2015) proposed that sensitive periods evolve when there is variation across ontogeny in (a) the availability of cues, (b) the informativeness of cues, (c) the fitness benefit of information, or (d) the fitness cost of plasticity. First we briefly describe each of these arguments. Then we discuss a model of mid-ontogeny sensitive periods, which we recently developed.

(a) A cue might only be available in some life stages and not in others, or be more likely to occur in some life stages than others. For instance, Kuzawa (2005) hypothesized that pregnant women transmit physiological signals to their fetus, which provide a summary of her lifetime nutritional experience, and which the fetus uses to predict its own postnatal nutritional environment. As this putative cue is only present inside the womb, people may only be sensitive to this cue during the fetal life stage. In a similar way, sensitivity to other cues may be limited to later life stages. For instance, courtship cues – e.g., being approached with sexual intent – are non-existent or extremely rare early in life and increase in frequency closer to puberty. Therefore, people’s sensitivity to these cues, and their use of such cues in guiding their reproductive strategies, might increase over the course of childhood. People apparently use the quality of courtship cues to estimate their own desirability as a mate – (unromantically) referred to as ‘mate value’ in the biological sciences – and then use this estimate to determine what attributes they expect in future mates (e.g., which value such a mate should have, which level of commitment to the relationship, which level of investment in shared offspring) (Conroy-Beam et al., 2016).

A cue might also become available, or increase in frequency, at the life stage in which animals first have to navigate the environment on their own, independently of their parents. A test case exists when individuals differ in the timing of this species-typical developmental milestone. For instance, consistent with the stress acceleration hypothesis, rodent pups that receive low levels of maternal care leave the nest at a younger age, and such fledging is accompanied by accelerated development of emotion circuits that enable learning about dangers that become relevant after fledging (Bath et al., 2016; Callaghan and Tottenham, 2016; Gee et al., 2013; Sullivan and Holman, 2010; for research showing parental modulation of learning in humans, see Tottenham et al., 2019; for a formal model exploring how a person’s attachment style in adulthood may be shaped by relationships early in life, see Chumbley and Steinhoff, 2019). In this case, individual variation in leaving the nest, a developmental milestone, is associated with individual variation in increased levels of plasticity. Applying this idea to human development, we may speculate that people experience a temporary increase in plasticity when they move from one environment to another (e.g., moving to a new school, or into a new neighborhood). We may also speculate that an increase in prediction error could be a mediating mechanism. The old environment was predictable, reducing the need for plasticity. After moving the individual might benefit from elevated levels of plasticity to learn the statistical structure of the new environment. Thus, by hypothesis, changes in the availability and frequency of cues – such as those occurring with a new set of experiences – might increase plasticity in cognitive functions that need to be adapted to aspects of the environment that are likely to have changed.

(b) Even if a cue is present throughout ontogeny, it may be more reliable in some life stages than others (Fawcett and Frankenhuis, 2015). For instance, in addition to courtship cues, people might use non-sexual social cues – such as receiving positive social attention – to estimate their mate value. Such social cues are present from birth, but their reliabilities as indicators of mate value might increase from infancy to adolescence; that is, social attention received by an infant (based on their ‘cuteness’) presumably conveys less information about mate value at puberty than social attention received by a prepubescent teen. A cue’s reliability sets an upper bound to how much can be learned from a cue. However, the amount of information an individual extracts from a cue also depends on her perceptual and cognitive abilities. This amount can increase over ontogeny if perceptual systems become more accurate as they mature, or if understanding a cue depends on acquired knowledge (e.g., a child may not understand a subtle form of social rejection used by adults). In such cases, mechanisms using the cue may increase their sensitivity to the cue over the course of ontogeny.

(c) Even if a cue provides the same amount of information throughout ontogeny, its potential to affect fitness might be higher at some life stages than others (Fawcett and Frankenhuis, 2015). For instance, an individual who receives a cue indicative of her mate value (e.g., courtship cues) will have more to gain from using this cue around puberty – when her future reproductive potential is high – than following menopause. Therefore, we may expect individuals to be more sensitive to such cues during adolescence compared with old age. In sum: adolescents might be particularly sensitive to social feedback for (at least) three different reasons: such feedback might be more available, more reliable, and have more scope to affect fitness.

(d) The costs of plasticity, like its benefits, might vary across ontogeny. These costs may include the energy invested in building, maintaining, and running the neural systems to perceive and use cues (Auld et al., 2010; DeWitt et al., 1998; Relyea, 2002). Although it has been challenging to document costs of plasticity empirically, there are convincing examples. For instance, fruit flies bred for enhanced (associative) learning ability evolve shorter life spans as a consequence. Their investment in plasticity thus trades off with somatic maintenance (Mery and Kawecki, 2003, 2004, 2005). When fruit flies are energetically starved (experimentally), their brain shuts down the formation of aversive long-term memories, which are costly to produce (Plaçais and Preat, 2013). Re-feeding starved flies, however, facilitates memory formation, showing that plasticity can be regained (Hirano et al., 2013). This flexibility suggests that plasticity (the ability to adjust development based on experience) does not change across ontogeny. Hence this example does not qualify as a sensitive period, according to our definition. However, the example does illustrate that plasticity may trade off with other energetically expensive activities. Accordingly, if all members of a species are low on resources at a particular life stage (e.g., salmon after having swum upstream to reproduce and die in their natal patch), we may speculate that natural selection favors a species-typical decline in plasticity at this life stage. The idea that plasticity is costly might initially seem at odds with the empirical finding that putting breaks on plasticity (e.g., perineuronal nets) is metabolically costly (Werker and Hensch, 2015), but it is not. If costly molecular mechanisms exist in order to regulate plasticity, they are a cost of plasticity; non-plastic organisms would not need such mechanisms.

### 4.2 Bridging evolutionary modeling and empirical paradigms

Formal models to date have assumed that cues are equally reliable in all time periods. We have recently developed a model in which the cue reliability varies across ontogeny (Walasek and Frankenhuis, 2019). In our model, individuals sample cues to the current conditions, while gradually – step-by-step, in each time period – tailoring their phenotypes to these conditions. We vary the cue reliability in three ways: cues may become more reliable over ontogeny (increasing), less reliable (decreasing), or first more reliable and then less reliable (triangular). To find out whether natural selection favors sensitive periods in mid-ontogeny, we evolve (optimal) developmental strategies in different environments (combinations of priors and cue reliability). Then we expose organisms following these strategies to experiences (cues) in order to observe optimal decisions and resulting developmental trajectories.

We use “study paradigms” that resemble those used in empirical research on sensitive periods to uncover plasticity. Studies of humans typically compare people who have been adopted (or have migrated) at different ages with each other, and with people who have not been adopted (or not migrated) (Mascie-Taylor, and Little, 2004; Pallier et al., 2003; Zeanah et al., 2011). Studies of non-human animals (e.g., rodents, birds) often use controlled experimental setups, such as *cross-fostering paradigms* in which animals are experimentally transferred at different times between different caregivers or patches (Breed and Moore, 2015), or *dose-dependent experience paradigms* that systematically vary the duration and amount of exposure to particular experiences (Groothuis and Taborsky, 2015).

We have explored similar types of paradigms in order to foster a bridge between theoretical and empirical studies of sensitive periods. We instantiate these paradigms by creating identical twins (clones) that are separated at different times during ontogeny and then exposed to different experiences. Next we measure the resultant differences in their phenotypes. If these differences are small, the developmental system had little plasticity at the time of separation; if it is large, it had much plasticity. We also vary the timing of separation. That is, we create and separate twins in each developmental time period. If twins separated early in life diverge more than twins separated later, there is a sensitive period early in life. If twins diverge most when separated mid-ontogeny, plasticity is highest in mid-ontogeny.

**Fig. 1:**
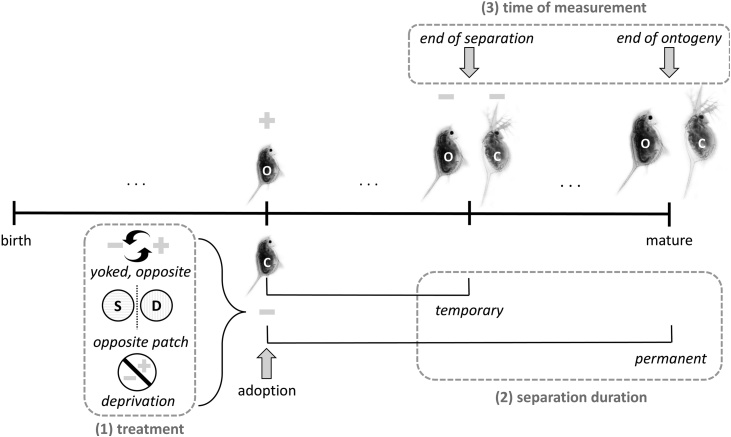
Measuring changes in plasticity across ontogeny. We separate twins (original, denoted as O, and clone, denoted as C) at different ages. We vary three dimensions: treatment, separation duration, and time of measurement. (1) *Treatment* refers to how the experiences of the original and clone differ during their separation. The clone might experience yoked, opposite cues; cues from the opposite patch; or deprivation. With yoked opposite cues, the clone always samples the opposite cue of the original: if the original samples a minus cue [−], the clone samples a plus cue [+]. With cues from the opposite patch, the clone samples a sequence of cues typical of the opposite patch: if the original tends to sample more minus cues, the clone tends to sample more plus cues. In our figure, the original and the clone are both in the dangerous patch (denoted as D), but the clone receives cues typical of the safe patch (denoted as S). With deprivation, the clone receives no cues or, equivalently, cues that are too noisy to extract information; thus preventing learning about the environment. (2) *Separation duration* refers to whether the separation of twins is permanent or temporary. Permanent separation occurs if twins experience different conditions from their separation until the end of ontogeny (maturity). Temporary separation occurs if twins are reunited before the end of ontogeny. (3) *Time of measurement* refers to when differences in the phenotypes of twins are measured. We measure differences in phenotypes of twins at two different time points: at the end of their separation and at the end of ontogeny. Our results show that different treatments tend to produce (qualitatively) similar patterns of plasticity. Our predictions are therefore similar for different treatments and for different measurement times used in empirical research. Copyright: we have used the images of Daphnia with permission from Dr. Linda Weiss (2018).

We also vary ‘how’ experiences differ between the twins during their separation (Fig. 1). Extreme divergence in experience occurs with *yoked, opposite cues*; whenever one twin samples a danger cue, the other samples a safe cue. This treatment is artificial; in empirical studies, it only occurs in controlled lab conditions. However, a milder form of divergence, *opposite patch cues*, more closely matches a situation in which twins are separated (through adoption or migration) into different conditions. Third, we explore *deprivation*, receiving no cues or cues that are too noisy to extract information from, which corresponds to sensory deprivation in lab conditions (Hubel and Wiesel, 1970) or exceptionally traumatizing real-world circumstances. For instance, children might have spent extended time in a dark enclosed space while in hiding during wartime (Wolf, 2007) or have grown up in very deprived orphanages (Kaler and Freeman, 1994).

We also compare ‘permanent’ versus ‘temporary’ separation. Permanent separation occurs when individuals experience different conditions from their separation until the end of ontogeny (maturity). In the real world, permanent separation might occur if one child is adopted and another is not, or if children are adopted into different homes. In lab conditions, permanent separation occurs in cross-fostering studies. Temporary separation might occur when siblings are separated (e.g., during a war or a natural disaster), and are later reunited within one home. In the lab, temporary separation occurs in dose-dependent experience studies, in which the experiences of individuals differ to a specific degree at a particular time. Finally, we measure differences in phenotypes of twins at two different time points: at the end of their separation and at the end of ontogeny.

In sum: we vary the timing of separation (age), the extent to which experiences differ (yoked, opposite cues; opposite patch; and deprivation), and whether separation is permanent or temporary. We then measure differences in phenotypes both at the end of their separation and at the end of ontogeny. Jointly, these treatments cover many paradigms used in empirical research.

Our results show that natural selection favors sensitive periods in mid-ontogeny in two conditions: if cues become more reliable over time, or if cues first become more reliable and then less reliable. If cues start out more reliable and become less reliable, sensitive periods are never favored in mid-ontogeny. These results are strikingly general across prior probabilities (under the assumption that cues are highly reliable in at least some time periods). Sensitive periods also look remarkably similar for increasing and triangular cue reliabilities. Perhaps in both cases organisms already tend to have good estimates of the state of the environment in mid-ontogeny (when cue reliabilities start declining in the triangular case and keep going up in the increasing case). Previous work has shown that if the cues are equally reliable and the environmental state is stable across ontogeny, sensitive periods evolve early in life (see Section 3.1). Integrating across models, we conclude that if cue reliabilities are either constant or decrease over ontogeny, sensitive periods evolve early in life; but if they increase over ontogeny (or are triangular), sensitive periods evolve mid-ontogeny. Our results depend on the study paradigm, but only as a matter of degree, not kind. This is good news: it means that the predictions of our model should hold across different treatments and times of measurement in empirical research.

### 4.3 ‘Belief-and-phenotype’ and ‘belief-only’ models

In some models, optimal decisions depend both on an organism’s phenotypic state (e.g., traits already developed) and on the information available to an organism about its environment, as a function of its evolved prior and the cues it has sampled during its lifetime (note: such estimates are often referred to as “beliefs”, even though conscious deliberation, or even psychological representation, is not necessarily involved). In these models, organisms that have identical beliefs might make different decisions because their phenotypes differ. Our model is of this kind. We call this a ‘belief-and-phenotype’ model. Other models assume a one-to-one mapping between beliefs and phenotypes. In these models, organisms do not have phenotypes, only beliefs. Individuals with the same beliefs thus always make the same decisions. We call such models ‘belief-only’ models.

Table 1 presents for a set of models of the evolution of sensitive periods whether each model includes only beliefs or beliefs and phenotypes, how plasticity is quantified, and information about the study paradigm. Some models have used an explicit paradigm (like the ones we just discussed) to expose changes in plasticity over time; others have not and require readers to infer the degree of plasticity. To compare findings and predictions across different models, it would be helpful if, when possible, researchers would use the same paradigms or explicitly describe their own paradigm.

**Table 1:** Comparison of formal models of sensitive periods. The first column lists the paper in which a model was published. The second column describes whether in this model an organism’s decisions depend only on its beliefs, or also on its phenotype. Note: the term “belief”, in this context, refers to the information available to an organism about its environment as a function of its prior and the cues it has sampled during its lifetime. It does not necessarily imply conscious deliberation or even psychological representation. The third column describes how plasticity is measured. The fourth column provides additional detail about the testing paradigm (e.g., when plasticity is measured).

Model	Phenotype (P) and/or Belief (B)	How is plasticity measured?	Study paradigm (if applicable)
Frankenhuis and Panchanathan (2011)	P&B	Number of cues sampled	
Fischer et al. (2014)	P&B	Phenotypic adjustment after each time period in response to a sampled cue, current phenotype, and current belief about the environmental state	Phenotypic adjustment is measured after each cue; a range of cue reliabilities is explored
Stamps and Krishnan (2014a)	B	Difference in beliefs after repeated exposure to the same cue	Each individual is exposed to the same cue four times; differences in beliefs are measured after each time period
Stamps and Krishnan (2014b)	B	Within-individual design: absolute difference in beliefs before and after exposure to a cue at different ages Sequential design: one individual is exposed to two different cues for an extended period of time	Difference in beliefs is measured after each cue Three cue reliabilities: high, low, and moderate levels of danger
English et al. (2016)	P&B	Within-individual effects of temporary food supplementation or deprivation during different time periods on phenotypes (age and size at maturity, reproductive success)	Extreme divergence (supplementation or deprivation); temporary treatment; plasticity is measured at the end of ontogeny
McNamara et al. (2016)	P&B	Phenotypic variance of a genotype is attributed to different sources of cues	
Panchanathan and Frankenhuis (2016)	P&B	Phenotypic divergence between simulated twins as a function of separation time	Extreme divergence between experiences (yoked opposite cues); permanent separation; plasticity is measured at the end of ontogeny
Stamps and Krishnan (2017)	B	Within-individual design: absolute difference between beliefs before and after exposure to a cue at different ages Replicate-individual design: absolute differences between beliefs after exposure to different cues at the same age (measured at different ages)	Difference in belief is measured after each cue; various patterns of cue reliabilities are explored

Stamps and Krishnan (2014a, 2014b, 2017) have usefully studied the effects of within-individual and replicate-individual designs. Within-individual designs measure plasticity as the difference in belief both before and after exposure to a cue. Replicate-individual designs measure the difference between beliefs of two organisms after each is exposed to a different cue. If such designs would be used in all future models, this could accelerate the development of an integrative theoretical framework of sensitive periods.

## 5 Gaps and future directions

We have described the tenets of evolutionary models of sensitive periods, insights they provide, predictions they make, and a selection of empirical research on humans and other animals. We now turn to gaps in the literature as well as future directions. We have already discussed the need for more formal models of the evolution of sensitive periods in mid-ontogeny. We have also stressed the need for formal modelers to use a consistent set of methods for exposing changes in plasticity across the life course, ideally matching commonly-used empirical paradigms, such as studies of adoption and migration, cross-fostering, and dose-dependent experience. We discuss four other future directions.

First, the field needs more models that explore environmental variation occurring within the lifetime of individuals. For instance, in our model of varying cue reliabilities (see Sections 4.2 and 4.3), experience varies quantitatively (i.e., more or less reliable cues), but not qualitatively (i.e., different kinds of experience). However, real animals often face different kinds of adaptive challenges and different types of information at different life stages (Bjorklund, 1997; Turkewitz and Kenny, 1982). Consider, for instance, human development in the first year of life: “the training sets for statistical learning develop as the sensorimotor abilities of the infant develop, yielding a series of ordered datasets for visual learning that differ in content and structure between time-points but are highly selective at each time-point. These changing environments may constitute a developmentally ordered curriculum that optimizes learning across many domains” (Smith et al., 2018, p. 325; see also Adolph and Hoch, 2019). Moreover, starting at birth, infants are active agents that scan their environments and select the objects and events they attend to (Gibson, 1988). Future models could explore the evolution of sensitive periods when the experiences of organisms vary qualitatively over ontogeny, either because individuals create a curriculum for learning (and how organisms do this might itself be under selection), or because the environmental state varies over ontogeny (e.g., seasonality). Which inputs inform experience at different times of life will depend on the statistical structure of the environment. Models of such inputs should therefore be informed by empirical measures of environmental statistics – in particular, cue reliability and autocorrelation of environmental states – known to be critical dimensions in the evolution of sensitive periods (Frankenhuis et al., 2019a).

Second, few models of the evolution of sensitive periods have explored the evolution of sequences of sensitive periods. Neuroscience suggests that such sequences help to build a well-structured brain. For instance: “Each succeeding large-scale region of cortex may […] be thought of as processing increasing orders of invariants from the stimulus stream, and passing either the invariant information extracted from the stream, or the residual information once the invariant is extracted, forward to other regions of the brain” (Shrager and Johnson, 1996, p. 1119). In this way, the organism incrementally learns about higher-order invariants and adapts to them. Perceptual ‘constraints,’ previously thought to be limitations, might actually facilitate this process, i.e., be adaptations. For instance, infants’ vision starting out blurry may help with basic-level category learning (French et al., 2002).

There are models of sensitive periods in neural development, which are often based on neural network architectures (Bullinaria, 2003; Ellefsen, 2013; Hurford, 1991; Kirby and Hurford, 1997; Seidenberg and Zevin, 2006). These models typically explore the effects of proximate factors and processes, such as neuromodulation, on sensitive period development. These models are able to generate, for instance, changes in plasticity that resemble those produced by neural processes (e.g., sequences of sensitive periods). The goal is typically to evaluate existing hypotheses, or to generate novel hypotheses, about proximate factors and processes involved in sensitive periods (e.g., how heterogeneity in experience might affect the ability to learn language; Seidenberg and Zevin, 2006). These models are not designed, however, to provide insight into the evolution of development. Future modeling could integrate both adaptive function and mechanism (e.g., exploring the evolution of mechanisms that produce progressive sequences of sensitive periods). Such modeling would fit the current agenda in evolutionary biology to better integrate mechanisms into optimality models of behavior (Frankenhuis et al., 2019b; Kacelnik, 2012; McNamara and Houston, 2009; Trimmer et al., 2012).

Third, the models we have discussed define plasticity as the ability to adjust phenotypes to environmental conditions based on experience. There are however other definitions plasticity, such as the capacity for reacting to a mismatch between supply and demand (Lövdén et al., 2010). Future modeling should explore the conditions in which natural selection favors sensitive periods when other notions of plasticity are employed. For instance, our definition does not distinguish between *flexibility*, the adaptive reconfiguration of existing abilities without structural change, and *plasticity*, lasting structural change in response to environmental inputs (Wenger et al., 2017; see also Fox et al., 2010). Future models could explore when it is adaptive for systems to exhibit changes in flexibility versus plasticity over the life course. Such models could incorporate the fact that structural changes can be beneficial, but also have the potential to be maladaptive by making the brain more vulnerable (Callaghan et al., 2019; Fuhrmann et al., 2015; Gee, 2016; Meyer and Lee, 2019; Opendak et al., 2017; Pattwell and Bath, 2017); for instance, when exposed to adverse experiences that are more extreme than human developmental systems have evolved to expect (Humphreys and Zeanah, 2015). Evolutionary models of sensitive periods have incorporated costs of plasticity (see Section 4.1). However, these costs are often fixed, rather than a function of the amount of plasticity (i.e., more plasticity implies a greater cost). It would be straightforward to incorporate vulnerability into models of the evolution of sensitive periods by making the cost of plasticity a continuous function of an organism’s level of plasticity.

Fourth, evolutionary models of sensitive periods to date assume that natural selection has equipped organisms with instructions for adaptive behavior; that is, organisms are born knowing which decisions are adaptive given their current phenotypic and/or belief states. This assumption is appropriate when modeling certain traits, such as the defensive armor that Daphnia (crustaceans) grow to protect against predation (Agrawal et al., 1999), which do not depend on feedback during an individual’s lifetime. There are many cases, however, where the development of phenotypes depends on learning from past behaviors (Snell-Rood, 2012). When organisms do not come equipped with instructions for adaptive behavior, but need to learn such instructions, a division of labor arises: natural selection shapes the learning mechanisms and the developing organism learns how to behave adaptively (Frankenhuis et al., 2019b). Recent models have started to examine how natural selection might shape the reinforcement learning mechanisms that enable organisms to learn adaptive behaviors (e.g., Dridi and Lehmann, 2015; Enquist et al., 2016; Singh et al., 2010; Sorg, 2011; Yeh et al., 2018). However, these models have to our knowledge yet to examine the evolution of sensitive periods in the learning of adaptive behavior. This would be a very exciting direction for future research. Moreover, organisms do not only learn specific adaptive behaviors (e.g., how to crack a nut), but also learn and select among broader decision-making strategies. For instance, depending on the level of control that an organism can exert over its environment (agency), it might calibrate its behavioral responses along a continuum ranging from proactive (‘What can I do in this environment?’) to reactive (‘What can this environment do to me?’) (Moscarello and Hartley, 2017). Future modeling should therefore explore the evolution of sensitive periods in the development of specific behaviors as well as broader decision-making strategies. This work may connect with recent models of meta-reinforcement learning, which examine when agents should compose meta-policies that can switch among a set of previously learned policies.

Fifth, our future directions so far have focused on ways in which evolutionary models can be made more relevant to research in cognitive developmental neuroscience. On the other side of the bridge, neuroscientists can help to foster synergies by including high-quality measurements, ideally longitudinally, of the physical and social environment experienced by individuals in studies on neural development. Evolutionary modelers can use such measurements to estimate environmental statistics, such as cue reliability and environmental autocorrelation (Frankenhuis et al., 2019a). Adaptation is essentially about the fit between individuals and their environments. Understanding adaptation, therefore, requires high-quality measurements of both. Large-scale longitudinal research projects that include detailed measurements of individuals’ environments (objective measures) and lived experience (subjective measures), as well as measurement of cognitive and neural development, hold particularly great promise for synergies with evolutionary modeling.

To end: evolutionary biologists have been interested in adaptive behavior and development ever since Charles Darwin proposed the process of natural selection. Only recently, however, evolutionary biologists have developed an explicit interest in adaptive changes in levels of plasticity over the life course. This trend fits perfectly with the long-lasting focus on sensitive periods in developmental cognitive neuroscience. The time is ripe, therefore, for stronger ties and novel synergies between these two exciting fields. We hope our paper will contribute a small step towards this goal.

## Funding

This research was supported by grants from the Netherlands Organization for Scientific Research (016.155.195), the James S. McDonnell Foundation (220020502), and the Jacobs Foundation (2017 1261 02) to WEF.

## Declaration of Competing Interest

We have no conflict of interest to declare.
